# Understanding the Dual Roles of CircHIPK3 in Tumorigenesis and Tumor Progression

**DOI:** 10.7150/jca.78090

**Published:** 2022-11-28

**Authors:** Zeyu Wei, Yijia Shi, Changning Xue, Mengna Li, Jianxia Wei, Guiyuan Li, Wei Xiong, Ming Zhou

**Affiliations:** 1 NHC Key Laboratory of Carcinogenesis, Hunan Cancer Hospital and The Affiliated Cancer Hospital of Xiangya School of Medicine, Central South University, Changsha, Hunan 410013, China.; 2 Xiangya Stomatological Hospital, Central South University, Changsha, Hunan 410078, China.; 3 Cancer Research Institute, Central South University, Changsha, Hunan 410078, China.; 4 The Key Laboratory of Carcinogenesis and Cancer Invasion of the Chinese Ministry of Education, Central South University, Changsha, Hunan 410078, China.

**Keywords:** circHIPK3, dual roles, differential expression, regulation mechanism, tumorigenesis

## Abstract

CircHIPK3 is a type of endogenous circular RNA, which contains a covalently closed circular structure and cannot encode protein or polypeptide. CircHIPK3 is unusually expressed in varieties of tumors and plays dual roles of tumor promotion or tumor inhibition in tumorigenesis and development of tumors by serving as the sponge for miRNA in multiple tumors. Here, we reviewed the differential expression, the dual functions, the regulation mechanism, and the network in a variety of tumors as well as the potential value for the diagnosis and treatment of tumors, which are of great significance for our comprehensive understanding of the roles and mechanisms of circHIPK3 in tumors.

## 1. Introduction

CircRNAs, a class of endogenous non-coding RNAs, feature covalently closed circular structures without a 5' cap, a 3' poly(A) tail or 5′ to 3′ polarity 1. Thus, circRNAs are highly stable and resistant to degradation by RNases. CircRNAs are mainly transcribed in the nucleus by RNA polymerase II 2, and both direct backsplicing and exon skipping can result in the formation of circRNAs 3. CircRNAs are widely found in eukaryotes and exhibit many types, tissue-specific expression, and conservation 4, 5. In addition, miRNAs can be regulated by circRNAs with one or more miRNA binding sites 6. Some circRNAs contain at least one internal ribosome entry site and open reading frame and thus can encode polypeptides or proteins 7. CircRNA homeodomain-interacting protein kinase 3 (circHIPK3) is derived from exon 2 of the HIPK3 gene which is located on chromosome 11p13 8. HIPK3 is flanked by long introns, and circHIPK3 is formed via these long introns with reverse complementary Alu repeats 3. CircHIPK3 is involved in the occurrence and development of a variety of diseases and pathological conditions, including diabetes mellitus 9, 10, pulmonary fibrosis 11, retinal dysfunction 9, cardiac fibrosis 12, 13, myocardial ischemia-reperfusion injury 14 and many others 15, 16. Besides, many cancers, such as colorectal cancer, gallbladder cancer, hepatocellular carcinoma, and lung cancer, have abnormal expression of circHIPK3. Increasing evidence indicates that circHIPK3 plays a crucial role in the initiation, development, and metastasis of multiple cancers. Therefore, circHIPK3 may contribute to diagnosis, therapy, and prognostic evaluation in cancers. Here, we review the differential expression, dual functions, regulatory mechanisms, and networks of circHIPK3 in a variety of tumors as well as its potential diagnostic and therapeutic value in cancers.

## 2. The dual roles of circHIPK3

CircRNAs are consistently abnormally expressed in some cancers, contributing to the functions involved in tumor promotion or suppression, including the regulation of cell survival, proliferation, migration, apoptosis, drug resistance, and so on. CircHIPK3 is notable for the differences in its expression profile, action mechanisms and biological functions in a variety of tumors. Therefore, circHIPK3 has dual roles in tumor promotion and inhibition in carcinogenesis and cancer development that depends on the type of cancer and the stage of tumor progression.

### 2.1 Oncogenic roles of circHIPK3

CircHIPK3 plays a mainly oncogenic role in most tumors. Among its functions, circHIPK3 promotes tumor development, progression, and metastasis in a variety of cancers, such as oral squamous cell cancer, nasopharyngeal cancer, gastric cancer, lung cancer, hepatocellular carcinoma, colorectal cancer, pancreatic cancer, prostate cancer, melanoma (Table 1). In addition, circHIPK3 facilitates the development of multidrug resistance, including resistance to temozolomide, oxaliplatin, gemcitabine, through different mechanisms.

#### 2.1.1 Promotion of carcinogenesis and tumor progression

CircHIPK3 is upregulated in varieties of cancers and can promote cancer cell proliferation, invasion as well as metastasis. There might be multiple mechanisms by which circHIPK3 plays oncogenic roles, leading to the establishment of different regulatory axes and networks.

##### 2.1.1.1 Oral Squamous Cell Carcinoma

Oral squamous cell carcinoma (OSCC) is the most familiar oral malignancy 17. Wang et al. showed that the expression level of circHIPK3 was high in OSCC, while deletion of circHIPK3 suppressed OSCC cell proliferation 18. Besides, Jiang W et al. 19 found that the overexpression of circHIPK3 can significantly promote EMT, proliferation, invasion of OSCC cells, and inhibit cell apoptosis *in vivo* and *in vitro*, which revealed that circHIPK3 may play an oncogenic role in OSCC occurrence and development. Moreover, circHIPK3 was found to perform its biological function in OSCC through binding to miR-124 18. Bi L et al. verified that circHIPK3 promoted the expression of YAP1 through binding to miR-381-3p, enhancing OSCC growth and development 20. In addition, Jiang W et al. demonstrated that circHIPK3 sponged miR-637 and regulated NUPR1/PI3K/AKT pathway, promoting cell proliferation and invasion of oral squamous cell carcinoma 19.

##### 2.1.1.2 Nasopharyngeal carcinoma

Nasopharyngeal carcinoma (NPC), influenced by multiple etiological factors, has a high incidence in China 21. Ke et al. verified that ectopic expression of circHIPK3 induced NPC cell proliferation, metastasis as well as invasion. Besides, E74-like ETS transcription factor 3 (ELF3) expression was upregulated by circHIPK3 via sponging miR-4288, therefore enhancing the malignant phenotype and exerting oncogenic effects in nasopharyngeal carcinoma. Therefore, circHIPK3 plays an oncogenic role in NPC through the circHIPK3/miR-4288/ELF3 regulatory axis 22.

##### 2.1.1.3 Gastric cancer

Gastric cancer (GC), one of the cancers with high incidence, is impacted by environmental and genetic factors 23, and the key environmental carcinogenic factor is *Helicobacter pylori* (HP) infection 24. Liu et al. found that circHIPK3 was upregulated in all GC tissues and cells, and positively associated with clinical TNM stage and poor overall survival in GC patients, while circHIPK3 promoted gastric cancer cell proliferation, invasion and metastasis by activating the Wnt/β-catenin pathway 25. Li et al. reported that circHIPK3 regulated the miR-876-5p/PIK3R1 axis to play an oncogenic role in malignant progression 26. Wei et al. showed that circHIPK3 post-transcriptionally increased the expression of brain-derived neurotrophic factor (BDNF) via sponging miR-107, facilitating the development of GC cells 27. Moreover, Cheng et al. speculated that circHIPK3 had a relationship with T stage in GC by negatively regulating the expression of miR-124 and miR-29b 28. Jin et al. found that circHIPK3 sponged miR-653-5p and miR-338-3p to promote NRP1 expression, enhancing the metastasis and invasion of GC cells 29. Yang et al. verified that circHIPK3 was bound to miR-637 to promote AKT1 expression, enhancing the growth of gastric cancer 30. Overall, these observations indicate that circHIPK3 plays an oncogenic role in GC and might be a crucial diagnostic and therapeutic target in GC.

##### 2.1.1.4 Lung cancer

Lung cancer, another malignancy, has the highest incidence and mortality worldwide. Non-small cell lung cancer (NSCLC) is a common histological type of lung cancer 31. Lu et al. found that circHIPK3 sponged miR-149, while miR-149 negatively regulated FOXM1. Therefore, circHIPK3 promoted the proliferation and malignant progression of NSCLC cells through the circHIPK3/miR-149/FOXM1 regulatory axis 32. Tian et al. showed that circHIPK3 ascended IGF1 expression through negatively regulating miR-379 expression, thus promoting NSCLC cell proliferation. Therefore, circHIPK3 improved NSCLC cell proliferation through the circHIPK3/miR-379/IGF1 axis 33. Chen et al. demonstrated that circHIPK3 was abundantly expressed and contributed to cell proliferation, metastasis as well as invasion in lung cancer. This group found that a lack of circHIPK3 suppressed the malignant behaviors of lung cancer cells. Moreover, circHIPK3, acting as an autophagy regulator, was correlated with the progression of lung cancer. In addition, it was confirmed that silencing of circHIPK3 significantly induced macroautophagy/autophagy through the miR-124-3p/STAT3/PRKAA/AMPKα signaling pathway 34. Hong et al. verified that circHIPK3 functioned as a sponge for miR-107 and upregulated BDNF, which was directly targeted by miR-107, to elevate tumor metastasis and proliferation 35. Gu et al. found that circHIPK3 sponged miR-381-3p to promote the malignancy of lung cancer through the AKT/mTOR pathway 36.

##### 2.1.1.5 Hepatocellular carcinoma

Hepatocellular carcinoma (HCC) is a histological type of liver cancer, which occupies 90% of primary liver cancers 37. By measuring its level in biopsy tissues of HCC patients, Chen et al. found that circHIPK3 negatively regulated miR-124. Besides, circHIPK3 promoted the malignant behaviors of HCC cells, whereas miR-124 suppressed these behaviors in HCC cells. Moreover, the overexpression of AQP3, a target of miR-124, promoted HCC cell proliferation, invasion as well as migration. Therefore, this research indicated that circHIPK3 modulated the expression of AQP3 by sponging miR-124, thus forming the circHIPK3/miR-124/AQP3 regulatory axis, which participated in the development and progression of HCC 38. In addition, Yu et al. found that circHIPK3 sponged both miR-124 and miR-506, thus increasing the expression of their mutual target PDK2, and the expression of PDK2 had positive correlations with tumor size, lymph node metastasis and the TNM stage in HCC 39. Multidrug-related protein 4 (MRP4) is related to drug transport. Hu et al. found that circHIPK3 bound to miR-124-3p as well as miR-4524-5p to enhance MRP4 expression 40. Li et al. showed that circHIPK3 facilitated ZEB2 expression via binding to miR-338-3p, enhancing the metastasis of HCC cells 41.

##### 2.1.1.6 Colorectal cancer

In recent years, the incidence of colorectal cancer (CRC) has increased and increases greatly with age 42. However, the number of youngsters who are diagnosed with colorectal cancer is increasing 43. Accumulating data suggest that the circHIPK3 expression level was obviously higher in CRC cells than in normal cells. Silencing of circHIPK3 was found to inhibit malignant behaviors and contribute to good prognosis. Formin like 2 (FMNL2) was identified as a critical downstream molecule of circHIPK3 to induce the migration and proliferation of colorectal cancer cells; circHIPK3 raised the expression of FMNL2 and promoted colorectal cancer cells proliferation, invasion and metastasis via binding to miR-1207-5p 44. Zeng K et al. showed that the binding of circHIPK3 to endogenous miR-7 can be increased to inhibit miR-7 activity, resulting in increased expression of the proto-oncogenes FAK, EGFR, IGF1R and YY1, therefore facilitating tumor development and progression. Moreover, the expression of circHIPK3 was regulated by c-Myb, a transcription factor. Therefore, this study demonstrated that the c-Myb/circHIPK3/miR-7 regulatory axis also acted as a certain role in the progression and development of CRC 45. Overall, these observations indicate that targeting the circHIPK3/miR-1207-5p/FMNL2 and c-Myb/circHIPK3/miR-7 axes may constitute a potential therapeutic strategy for CRC.

##### 2.1.1.7 Pancreatic cancer

Pancreatic cancer (PC), a malignancy originating from pancreatic tissue, is generally characterized by poor prognosis 46. CircHIPK3 was found to be upregulated in PC cells and biopsy tissues, and the knockdown of circHIPK3 led to inhibition of the tumorigenesis and metastasis of PC cells. Moreover, circHIPK3 was found to target RASSF1 through binding to miR-330-5p, leading to boost malignant progression and metastasis of PC cells. Therefore, targeting the circHIPK3/miR-330-5p/RASSF1 axis might be a potential diagnostic and therapeutic strategy for PC 47.

##### 2.1.1.8 Prostate cancer

Prostate cancer (PCa), a common type of cancer in men 48, is more likely to be related to age than to other risk factors 49. Cai et al. clarified that the overexpression of circHIPK3 promoted prostate cancer cell proliferation, invasion as well as migration. ADAM17, one of the members of the ADAM family, was found to be upregulated and to have the same effect as circHIPK3. Moreover, circHIPK3 was found to perform as a tumor-promoting factor through modulating ADAM17 expression through binding to miR-338-3p 50. Cdc25b and Cdc2 were also found to be targeted by miR-338-3p and involved in the malignant behaviors of PCa cells 51. Besides, Chen D et al. demonstrated that circHIPK3 sponged miR-193a-3p and regulated MCL1 expression in PCa, promoting prostate cancer cell proliferation, migration, and invasion of PCa cells *in vitro* and tumor growth *in vivo* 52. Liu et al. reported that the expression of circHIPK3 had a positive relationship with MTDH, which was targeted by miR-448, promoting the progression of PCa 53. Moreover, Tang et al. found that circHIPK3 enhanced PCa progression through interacting with miR-212 to upregulate BMI-1, suggesting that circHIPK3 acted as a tumor promoter 54.

##### 2.1.1.9 Melanoma

Melanoma is a dangerous type of cancer that frequently occurs on the skin 55, 56. Zhu et al. reported that compared to normal skin tissues and cell lines, melanoma tissues and cell lines exhibited high level of circHIPK3 and low level of miR-215-5p. Moreover, circHIPK3 overexpression enhanced cell proliferation and tumor development. However, overexpression of miR-215-5p reversed the effects of circHIPK3 and inhibited the malignant behaviors of melanoma cells. YY1, a transcription factor, was targeted by miR-215-5p and had a negative effect on apoptosis. Therefore, circHIPK3 promoted the progression of melanoma via the circHIPK3/miR-215-5p/YY1 axis 57.

##### 2.1.1.10 Glioma

Gliomas are a heterogeneous group of brain tumors with different biological and clinical features. Besides, glioma subtypes and grades are typically defined by histological features 58. Gliomas are the most common primary intracranial tumors; they are originated from neuroepithelial cells and account for 40-50% of brain tumors. Jin et al. verified that circHIPK3 exhibited a high expression level in glioma cells and that there was a positive link between circHIPK3 overexpression and tumor development and poor prognosis in glioma. The results of functional investigation implied that circHIPK3 has a biological role in promoting the malignant progression of glioma cells. In addition, miR-654 was found to be sponged by circHIPK3, while Insulin Growth Factor-2 Binding Protein 3 (IGF2BP3) was found to be aimed by miR-654. According to CCK8 and transwell assays, IGF2BP3 overexpression was found to facilitate the malignant behaviors of glioma cells. Therefore, by binding to miR-654, circHIPK3 modulated the level of IGF2BP3 to perform as a tumor-promoting factor 59. Furthermore, miR-124-3p was identified to act as a tumor suppressor through targeting the gene serine/threonine kinase WEE1. Moreover, circHIPK3 bound to miR-124-3p to improve the development of glioma cells 60. Liu et al. demonstrated that circHIPK3 sponged miR-124 and promoted CCND2 expression, in turn enhancing glioma cell proliferation, migration and invasion 61. Hu et al. verified that STAT3 was the target gene of miR-124-3p and the overexpression of circHIPK3 promoted STAT3 expression via sponging miR-124-3p, thus promoting glioma cell proliferation and invasion 62. Therefore, circHIPK3 promotes the malignant progression of glioma, which might be a potential diagnostic and therapeutic strategy for glioma.

##### 2.1.1.11 Cervical cancer

Cervical cancer (CC), a malignant tumor, is common in women worldwide 63. Qian et al. found that the circHIPK3 level was increased but miR-338-3p was downregulated in CC. Silencing of circHIPK3 suppressed cervical tumorigenesis and CC metastasis. Mechanistically, circHIPK3 upregulated hypoxia-inducible factor-1α (HIF-1α) through binding to miR-338-3p, thus promoting tumor progression in CC. Therefore, the circHIPK3/miR-338-3p/HIF-1α pathway played a critical role in the carcinogenesis of CC 64. Wu et al. illustrated that circHIPK3 overexpression led to the upregulation of Fibroblast growth factor 2 (FGF2) expression and vice versa. In summary, circHIPK3 regulated FGF2 expression in CC cells via sponging miR-485-3p, which targets FGF2, thus contributing to tumor progression in CC 65.

##### 2.1.1.12 Cholangiocarcinoma

Cholangiocarcinoma (CCA), a malignancy of the biliary tract, harbors the feature of cholangiocyte differentiation 66, 67. The overexpression of circHIPK3 was found to enhance the malignant behaviors of CCA cells through binding to miR-637. In addition, lymphocyte antigen-6E (LY6E) was demonstrated to be a direct target of miR-637. Therefore, circHIPK3 positively regulated CCA cell proliferation and development through the circHIPK3/miR-637/LY6E axis 68.

##### 2.1.1.13 Gallbladder cancer

Gallbladder cancer (GBC) is a type of hepatobiliary malignancy derived from the mucosal lining of the gallbladder 69. Kai et al. indicated that circHIPK3 exhibited a high expression level in gallbladder cancer biopsy tissues and cells. CircHIPK3 was found to facilitate gallbladder cancer cell proliferation via inhibiting the activity of miR-124 and enhancing rho-associated protein kinase 1(ROCK1) and cyclin dependent kinase 6 (CDK6) expression. Moreover, both ROCK1 and CDK6 were identified as direct targets of miR-124 and had a negative correlation with miR-124. Therefore, circHIPK3, as a miR-124 sponge, was associated with negative modulation of miR-124 expression and positive modulation of ROCK1 and CDK6 expression, contributing to promoting gallbladder cancer progression through the miR-124/ROCK1 and miR-124/CDK6 regulatory axes 70.

##### 2.1.1.14 Esophageal squamous cell carcinoma

Esophageal squamous cell carcinoma (ESCC) accounts for approximately 90% of esophageal cancers. There are many causes of ESCC, including cigarette smoking, heavy alcohol consumption and genetic factors. It was found that circHIPK3 was highly expressed in ESCC tissues and cell lines, and its expression level had a positive correlation with the TNM stage. Moreover, circHIPK3 enhanced ESCC xenograft tumor growth, indicating that circHIPK3 acted as an oncogenic role in ESCC. Furthermore, circHIPK3 suppressed miRNA-599 expression and upregulated c-MYC expression. Mechanistically, circHIPK3 was identified to promote ESCC development through the miR-599/c-MYC axis 71. Besides, Yao et al. found that circHIPK3 facilitated AKT serine/threonine kinase 3 (AKT3) expression by sponging miR-124, thus forming the circHIPK3/miR-124/AKT3 axis to promote ESCC cell proliferation, migration, and epithelial-mesenchymal transition 72.

##### 2.1.1.15 Breast cancer

Breast cancer (BC) is a common malignancy in females. Qi L et al. found that circHIPK3 sponged miR-326 to enhance breast cancer cell proliferation, migration, and invasion 73. In addition, the results of a study by Chen et al. implied that circHIPK3 facilitated HMGB1 expression through binding to miR-193a, establishing the miR-193a/HMGB1/PI3K/AKT signaling axis, which enhanced the progression of BC cells 74. Shi et al. demonstrated that circHIPK3 enhanced MTDH expression via sponging miR-124-3p in endothelial cells (ECs), thus establishing the circHIPK3/miR-124-3p/MTDH signaling axis to enhance tube formation of BC cells 75.

##### 2.1.1.16 Thyroid cancer

Thyroid cancer (TC) is a common endocrine malignancy. Shu et al. indicated that circHIPK3 positively modulated RAB23 expression via binding to miR-338-3p, which contributed to enhancing the proliferation and invasion of TC cells 76.

##### 2.1.1.17 Chronic myeloid leukemia

Chronic myeloid leukemia (CML), a type of hematological malignancy, is related to certain hematopoietic cells. Feng et al. found that circHIPK3 sponged miR-124, which exhibited a decreased level in serum samples from CML patients. CircHIPK3 may regulate B4GALT1, which was targeted by miR-124, and played a vital oncogenic role by suppressing miR-124 expression 77.

#### 2.1.2 Promotion of multidrug resistance in a variety of tumors

##### 2.1.2.1 Temozolomide

Temozolomide (TMZ), an alkylating agent, is the most effective first-line chemotherapeutic drug for patients with glioblastoma. However, TMZ-induced chemotherapy resistance is the main factor leading to tumor recurrence and patient death. It was reported that circHIPK3 was upregulated in TMZ-resistant glioma cells. In addition, miR-524-5p was found to be sponged by circHIPK3, and KIF2A was found to be targeted by miR-524-5p. The suppression of KIF2A could enhance TMZ sensitivity and apoptosis to inhibit the proliferation and development of TMZ-resistant glioma cells, and the silencing of circHIPK3 also had the same effects 78. In addition, Han et al. proposed some new ideas. They found that miR-421, which was sponged by circHIPK3, was downregulated in TMZ-resistant glioma cells, while circHIPK3 was up-regulated. Besides, ZIC5 was targeted and regulated by miR-421, leading to the reversal of the antitumor effect of miR-421 79. In conclusion, circHIPK3 promoted glioma development as well as drug resistance through the miR-524-5p/KIF2A and miR-421/ZIC5 regulatory axes.

##### 2.1.2.2 Oxaliplatin

Accumulating data have implied that the circHIPK3 level is increased in patients with colorectal cancer (CRC) who exhibit chemoresistance. Zhang et al. indicated that the high expression of circHIPK3 in recurrent CRC tissues, was related with tumor size, tumor metastasis, and survival. Moreover, the overexpression of circHIPK3 promoted oxaliplatin resistance via inhibiting autophagy. Mechanistically, circHIPK3 increased the expression of STAT3 and initiated the downstream Bcl-2/beclin1 signaling pathway through binding to miR-637 80.

##### 2.1.2.3 Gemcitabine

Gemcitabine (GEM) is a novel antitumor pyrimidine analog. It was reported that the ectopic expression of circHIPK3 not only enhanced pancreatic cancer cell proliferation, invasion, and EMT but also led to resistance to gemcitabine. Mechanistically, circHIPK3 upregulated RASSF1 through sponging miR-330-5p, which resulted in malignant behaviors in pancreatic cancer cells, including resistance to GEM 47.

##### 2.1.2.4 Paclitaxel

Resistance to paclitaxel (PTX) weakens the effect of breast cancer therapy. CircHIPK3 plays a crucial role in PTX-resistant BC cells. Ni et al. inferred that the knockdown of circHIPK3 decreased HK2 expression through a reduction in binding to miR-1286, thus promoting PTX sensitivity in BC cells 81.

##### 2.1.2.5 Trastuzumab

Resistance to trastuzumab is an obstacle to successful breast cancer therapy. Zhang et al. found that the silencing circHIPK3 restrained trastuzumab resistance in breast cancer cells and that the upregulation of circHIPK3 expression promoted RNF11 expression via sponging of miR-582-3p, thus promoting trastuzumab resistance in drug-resistant breast cancer cells 82.

### 2.2 The functions of circHIPK3 as a tumor suppressor

Although circHIPK3 frequently contributes to the promotion of malignant behaviors in tumors, circHIPK3 can also suppress tumor progression in certain tumor types, which demonstrates its dual roles. Accumulating data have shown that circHIPK3 plays a tumor suppressive role in bladder cancer (Table 1).

## Bladder cancer

Bladder cancer is a type of cancer that forms in bladder tissues. Transitional cell carcinoma accounts for the majority of bladder cancers, and other types, including squamous cell carcinoma and adenocarcinoma, develop in the inner lining of the bladder. CircHIPK3 has always been reported to be a tumor suppressor gene with downregulated expression in bladder cancer. Li et al. indicated that circHIPK3 inhibited migration, invasion, and angiogenesis of bladder cancer cells *in vitro* and suppressed bladder cancer growth and metastasis *in vivo* via directly sponging miR-558. Moreover, heparanase (HPSE) was found to be targeted and negatively regulated by miR-558, whose expression was negatively related to the expression of circHIPK3. Therefore, circHIPK3 inhibited the growth, invasion, and lymph node metastasis of bladder cancer through the miR-558/HPSE regulatory axis 83. Furthermore, the overexpression of circHIPK3 in bladder cancer cells reduced the IC50 of gemcitabine and promoted gemcitabine cytotoxicity in T24/gem and J82/gem cells, thereby increasing the sensitivity of bladder cancer cells to gemcitabine 84.

### 2.3 Dual functions of circHIPK3 within the same tumor

As indicated above, circHIPK3 exerts dual roles of promotion or inhibition of tumorigenesis and tumor progression in different types of tumors, however, circHIPK3 was also found to present two kinds of different expression patterns and biological functions even within the same type of cancer in recent years, such as in renal carcinoma, osteosarcoma, ovarian cancer (Table 2). These results seem to be somewhat contradictory. Of course, first of all, the differences in detection results caused by the insufficiency of experimental operation must be eliminated. At the same time, the size of samples must be expanded and the experiments must be repeated several times to ensure the objectivity of the experimental results. On the other hand, we must objectively admit the rationality and scientificity of such results and carefully analyze the reasons for presenting these results. In terms of expression pattern, we believe this may be attributed to the complexity of circRNA production and upstream regulation, because the structural variation of circRNA encoding gene and regulatory changes in transcription level, splicing mode and RNA stability may lead to the differences in circRNA production and expression. At the same time, due to the differences in individual genetic susceptibility, environmental factors, and lifestyle of tumor patients, circRNA expression in different regions, populations and individuals of the same tumor type may vary to a certain extent. Of course, this does not mean that all circRNAs have dual expression patterns and functions within a type of tumor, and circHIPK3 may be just a particular case of the above. As for the difference of circHIPK3 biological function with the same tumor, after eliminating the non-authenticity resulting from experimental methods and artificial deviation, we believe that the choice of tumor cell lines is the important cause leading to the different biological function, because the function of some genes including circRNAs in different cell lines of the same kind of tumor types may be completely different.

#### 2.3.1 Renal cancer

Renal cancer is a malignant tumor that originated from the urinary tubule epithelial system of the renal parenchyma. The number of people diagnosed with renal cancer has increased to date 85, and older people are especially affected 86. Among the subtypes of renal cell carcinoma, clear cell renal cell carcinoma (ccRCC) is the most common 87. Lai et al. demonstrated that there were high circHIPK3 expression levels in renal cancer (RC) tissues and cells and that loss of circHIPK3 inhibited RC cell proliferation, migration, and invasion. In addition, circHIPK3 was found to sponge miR-485-3p, thus promoting the progression of RC cells 88. Han et al. clarified that circHIPK3 modulated the miR-508-3p/CXCL13 axis to accelerate tumor growth in ccRCC 89. These results demonstrated that circHIPK3 was upregulated in RC and exerted an oncogenic role in RC. However, there was also a contradictory report that circHIPK3 was down-regulated in ccRCC cells and that ectopic expression of circHIPK3 effectively suppressed ccRCC cell proliferation, invasion, and migration and xenograft tumor growth by sponging miR-637 in ccRCC, which seems that circHIPK3 may function as a tumor suppressor through targeting miR-637 in ccRCC 90.

#### 2.3.2 Osteosarcoma

Osteosarcoma (OS), one of the most common bone malignancies, occurs commonly in adolescents and individuals under the age of 20. Wen Y et al. reported that circHIPK3 was upregulated in OS tissue and cell lines, while the knockdown of circHIPK3 suppressed OS cell proliferation, migration, and invasion, suggesting that circHIPK3 expression was useful for the prediction of osteosarcoma. Moreover, circHIPK3 was identified as a sponge of miR-637, and histone deacetylase 4 (HDAC4) was the downstream target of miR-637. Therefore, circHIPK3 enhanced the malignant behaviors of OS cells by regulating the miR-637/HDAC4 axis 91. In addition, Huang Z et al. found that circHIPK3 facilitated cell migration, cell invasion, and tumor growth by inhibiting the miR-637/STAT3 axis 92. These results support that circHIPK3 was highly expressed in OS tissues and promoted tumor progression through miR-637/HDAC4 and miR-637/STAT3 axis in OS. However, Xiao-Long M et al. found that circHIPK3 was down-regulated in the OS cell lines, tissues and plasmas, and negatively correlated with Enneking stage, lung metastasis and poor prognosis of OS patients, and the overexpression of circHIPK3 significantly suppressed OS cell proliferation, migration and invasion *in vitro* 93.

#### 2.3.3 Ovarian cancer

Ovarian cancer, a malignancy occurring in women, is more commonly diagnosed in the elderly population 94, 95. Some data revealed that circHIPK3 exhibited a high expression level in ovarian cancer (OC). Meanwhile, there was a positive link between high circHIPK3 expression and invasion, poor prognosis and high mortality in OC 96. Moreover, Zhou et al. found that circHIPK3 enhanced the proliferation of OC cells by sponging miR-7 thus promoting VEGF expression 97. However, there was a report of contradictory results. Teng et al. further found circHIPK3 was downregulated in ovarian cancer via RNA sequencing, the knockdown of circHIPK3 facilitated the proliferation, invasion, and migration and suppressed the apoptosis of OC cells, showing that circHIPK3 acted as a tumor suppressor in ovarian cancer 98. Of course, these results need to be replicated and verified further.

## 3. Mechanism and network of circHIPK3 in tumor behaviors

CircHIPK3 plays critical roles in tumor progression and drug resistance mainly through the following mechanisms, including its upstream and downstream regulators, constituting a functional regulatory network, and determining the expression pattern and biological function in different tumors.

### 3.1 The upstream regulation of circHIPK3

**Figure 1:**
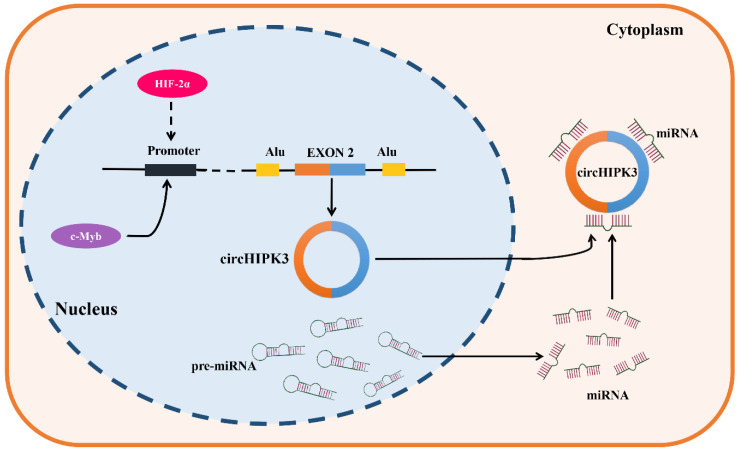
** Generation and upstream regulation mechanism of circHIPK3.** CircHIPK3, a sort of circRNAs which is derived from the exon 2 of HIPK3 gene, locates in the chromosome 11p13. C-Myb was confirmed as a transcription factor of circHIPK3, which could bind the promoter region and promote the transcription activity and expression of circHIPK3 in CRC. In addition, HIF-2α was found to positively regulate the expression of circHIPK3 in GC under a hypoxic microenvironment, indicating that circHIPK3 is a potential transcription regulation target of HIF-2α.

CircRNAs are formed by backsplicing of pre-mRNAs. They are usually subject to diverse upstream regulatory effects, including transcriptional regulation, epigenetic modification, and RNA stability 99. CircHIPK3 was found to be upregulated in most types of tumors, such as lung cancer, oral squamous cell cancer, gastric cancer, colorectal cancer, prostate cancer, gallbladder cancer, nasopharyngeal cancer and chronic myeloid leukemia, but down-regulated in bladder cancer. It is noteworthy that circHIPK3 was found to present the dual expression pattern in osteosarcoma, ovarian cancer, and clear cell renal cell carcinoma. These results demonstrated the complexity of circHIPK3 upstream regulation. C-Myb is a critical oncogenic transcription factor, which was reported to be involved in the upstream regulator of circHIPK3 in CRC, the results of a ChIP assay implied that c-Myb can bind to the promoter region of circHIPK3, and the overexpression of c-Myb significantly enhanced the promoter activity and expression of circHIPK3, therefore these results support that circHIPK3 was a direct target of c-Myb in CRC 45. In addition, the knockdown of HIF-2α but not HIF-1α decreased the expression of circHIPK3 in GC under a hypoxic microenvironment, and a strong positive correlation was also verified between HIF-2α and circHIPK3 in GC samples, indicating that circHIPK3 was a potential target of HIF-2α. Indeed, it still needs to be verified whether circHIPK3 is directly upregulated by HIF-2α transcription or is upregulated by another HIF-2α target gene. Further study is warranted in the future 29. Objectively, the upstream regulatory and generation mechanisms of circHIPK3 have been poorly studied (Figure 1), so its upstream mechanisms are also lacking. Therefore, we hope more studies on the upstream regulation of circHIPK3 will be followed to improve it.

### 3.2 The downstream regulation of circHIPK3

CircRNAs compete with miRNAs depending on their miRNA response elements (MREs), thus affecting miRNA target gene expression. Therefore, circRNAs can work as miRNA sponges to regulate mRNA expression at the post-transcriptional level, and thus circRNAs function as competing endogenous RNAs (ceRNAs) to regulate the expression of target mRNA 100. As indicated above, circHIPK3 can not only play an oncogenic role in in most tumor types including oral squamous cell carcinoma, hepatocellular carcinoma, nasopharyngeal carcinoma, gastric cancer, lung cancer, pancreatic cancer, prostate cancer, melanoma, glioma, cervical cancer, cholangiocarcinoma, gallbladder cancer, esophageal squamous cell carcinoma, colorectal cancer and so on, but also work as a tumor suppressor in bladder cancer (Table 1). Most importantly, circHIPK3 was reported to present dual expression patterns and functions within the same tumor, such as osteosarcoma, ovarian cancer, and clear cell renal cell carcinoma (Table 2). All of these results suggested the complexity of circHIPK3 function and downstream mechanism. As we know that circHIPK3 is just a regulation molecule, where the type of downstream target molecules of circHIPK3 determines its function as an oncogene or tumor suppressor in different types of tumors even different cell lines with the same tumor. Several miRNA response elements have been found in the RNA sequence of circHIPK3, and each of the targeted miRNAs targets one or more different genes. CircHIPK3 sponges several miRNAs and increases the expression of their target genes expression by removing the inhibition imposed by these miRNAs. Therefore, the role of circHIPK3 depends on the corresponding miRNA/target regulatory axes and tumor type (Table 1 and 2). For example, circHIPK3 sponged miR-4288 and regulated the miR-4288/ELF3 regulatory axis to promote the proliferation, migration and invasion of NPC cells, thus functioning as an oncogene in NPC 22. At the same time, circHIPK3 regulated the expression of HPSE by sponging miR-558, thus functioning as a tumor suppressor in bladder cancer through the circHIPK3/miR-558/HPSE regulatory axis 83. In addition, circHIPK3 was found to play an oncogenic role via miR-508-3p/CXCL13 in renal cancer 89, but exerted a tumor suppressor role by sponging miR-637 in other renal cancer cell lines 90. These results seem to be somewhat contradictory. After eliminating the non-authenticity resulting from experimental methods and artificial deviation, we think that the choice of tumor cell lines is also the critical cause leading to the different biological functions, because the functions of some genes including circRNAs in different cell lines with the same tumor may be completely different.

It has been reported that some circRNAs can interact with RNA-binding proteins (RBPs) and compete with the parental mRNA to modulate gene expression at the transcriptional and posttranscriptional levels. Although there is no evidence indicating that circHIPK3 can directly bind to RBPs and regulate their expression, it regulates the expression of RBPs through the ceRNA mechanism. For example, Pereira found that the circHIPK3/miR-107/BDNF/LIN28 axis was related to chemoresistance in gastric cancer. CircHIPK3 can inhibit the expression of miR-107 via sponging it, which promoted the activity of brain-derived neurotrophic factor (BDNF). In addition, BDNF can enhance the activity of LIN28. CircHIPK3 can bind to LIN28A and LIN28B. CircHIPK3 can enhance the function of LIN28. Furthermore, a high LIN28 protein level was found to regulate the expression of miR-107 and lead to the resistance of gastric cancer to oxaliplatin, fluorouracil, doxorubicin, paclitaxel and so on 101.

**Figure 2:**
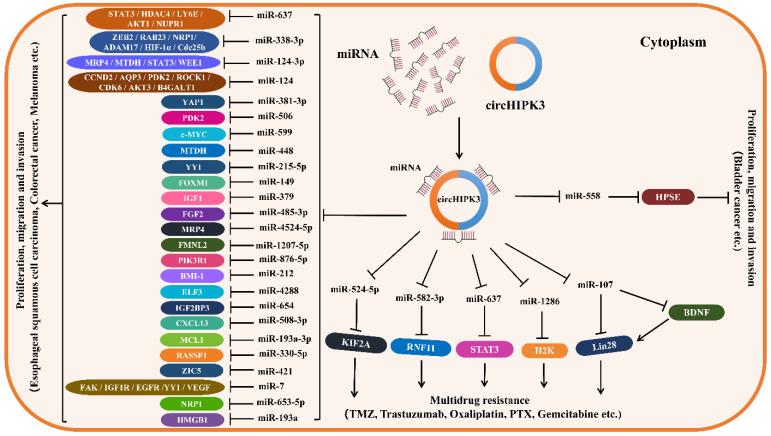
** Downstream regulatory network of circHIPK3.** CircHIPK3 is frequently abnormally expressed in some types of cancers, playing an oncogenic role in most cancers, such as lung cancer, oral squamous cell cancer, gastric cancer, colorectal cancer, and so on. CircHIPK3 also plays an anti-tumor role in bladder cancer. CircHIPK3 can act as a miRNA sponge to regulate the expression of mRNA at the post-transcriptional level, making contributions to the functions of promoting or restraining cancers. CircHIPK3 can also regulate the expression of RBP through ceRNA mechanism and compete with target mRNA to modulate gene expression at transcriptional and post-transcriptional levels.

Since circHIPK3 regulates the levels of related target genes by sponging various miRNAs (miR-124, miR-637, miR-338-3p, miR-107, miR-29b, etc.), thus forming circHIPK3-miRNA-mRNA regulatory axes to be involved in the tumorigenesis and tumor progression. Moreover, circHIPK3 can regulate some RBPs, such as KIF2A, BDNF and LIN28, by sponging miRNAs to increase drug resistance in some cancers. Therefore, we established the downstream regulatory networks of circHIPK3, which are thought to be involved in tumor progression and drug resistance (Figure 2). In addition, we summarized the downstream pathways of circHIPK3 involved in different tumors based on its targets and related signal pathways reported in some literature (Table 3), of them PI3K/AKT, Wnt/β-catenin were the critical signaling pathways most affected by circHIPK3, which is beneficial to deepen the understanding of the downstream mechanism of circHIPK3 in tumors. Certainly, the present research on the downstream mechanism of circHIPK3 is limited to acting as a miRNA sponge regulating the expression of downstream target genes, which is actually very deficient. Whether circHIPK3 can function as a protein or RNA scaffold to bind to RNA-binding protein, or encode polypeptide has not been reported in any literature so far, which needs to be further explored in future studies.

## 4. Potential value of circHIPK3 in the clinical diagnosis and treatment of cancers

CircRNAs have the characteristics of high stability, conservation, and tissue-specific expression, suggesting that some circRNAs might have potential use as biomarkers for the clinical diagnosis of cancers 1. CircHIPK3 has been found to be highly expressed in multiple cancers, such as lung cancer, oral squamous cell cancer, gastric cancer, colorectal cancer, prostate cancer, gallbladder cancer, nasopharyngeal cancer and chronic myeloid leukemia, and its expression level is positively associated with advanced TNM stage and poor prognosis in patients with these cancers. Conversely, circHIPK3 has been found to be expressed at low levels in bladder cancer, clear cell renal cell carcinoma and ovarian cancer, and its expression level is negatively associated with advanced TNM stage and poor prognosis in patients with these cancers. All of these results suggest that circHIPK3 might serve as a potential biomarker for the clinical diagnosis of these cancers. For example, circ_0000190 was identified as a novel non-invasive biomarker for the diagnosis of gastric cancer, which had better sensitivity and specificity than those of commonly used biomarkers such as CEA and CA19-9 102. Chen et al. demonstrated that when the ratio of circHIPK3 to linear HIPK3 (C:L ratio) was > 0.49, it could be used as a biomarker of poor prognosis, especially for patients with advanced NSCLC 34. CircHIPK3 was significantly less abundant in serum extracellular vesicle (sEV) from glioblastoma multiforme (GEM) patients with respect to unaffected controls (UC) (fold-change (FC) of -1.92) and grade 3 glioma (GIII) (FC of -1.4), and receiver operating characteristic curve (ROC) analysis allowed us to distinguish GBM from UC (area under the curve (AUC) 0.855 (0.704 to 1.000), with a 95% confidence interval (CI)), which indicated that sEV-derived circHIPK3 could serve as a good diagnostic biomarker for GBM 103.

As indicated above, circHIPK3 has dual roles in tumor promotion and inhibition in the carcinogenesis and tumor development, and these roles depend on the type of cancer and the stage of tumor progression. CircHIPK3 has been found to be highly expressed in tumor tissues compared with normal tissues and exerted oncogenic roles in many types of cancers, while knockdown of circHIPK3 reversed the malignant phenotypes of these cancers. Therefore, we could interfere the expression of circHIPK3 in these tumors by introducing the lentivirus or nanoparticles-encapsulated sh-circHIPK3 into the specific tumor tissues, thus to inhibit the tumor growth and malignant progression. Conversely, circHIPK3 has been found to be expressed at low levels in the tumor tissues compared with the corresponding normal tissues and to play tumor suppressive roles in other cancers, such as bladder cancer, clear cell renal cell carcinoma and ovarian cancer, while the overexpression of circHIPK3 rescued the malignant phenotype of these cancers. Accordingly, we could rescue the expression of circHIPK3 by introducing its expression vector into the tumor sites, thus to inhibit the tumor growth and progression. In addition, circHIPK3 can be used as a sensitizer to promote sensitivity to gemcitabine in bladder cancer patients. Therefore, targeting circHIPK3 might be a promising therapeutic strategy for cancers, and the design of small molecule drugs, antisense oligonucleotides, and other methods to interfere with circHIPK3 should be a potential research direction for drug development in the future.

## 5. Conclusion

CircHIPK3 is a critical circRNA with dual functions in tumor development and progression. It can not only act as an oncogene to facilitate cell proliferation, migration, invasion and xenograft tumor growth and metastasis as well as the drug resistance in most tumors but also play a tumor-suppressive role by inhibiting cell proliferation, invasion and xenograft tumor growth and metastasis in a few cancers. In addition, the expression of circHIPK3 is different in different cancer types, which is correlated with the TNM stage and poor prognosis. As an oncogenic role, overexpressed circHIPK3 is positively correlated with the TNM stage and tumor size, such as esophageal squamous cell carcinoma. However, high level of circHIPK3 expression in other cancer types is negatively related to cancer grade and lymph node metastasis when it plays a tumor suppressor role, such as bladder cancer. Moreover, circHIPK3 has been verified to function as a miRNA sponge to mediate tumor progression and drug resistance through circHIPK3-miRNA-mRNA regulatory axes, and other mechanisms need to be further defined. Based on the expression profile and functions of circHIPK3 in carcinogenesis and drug resistance, circHIPK3 might be a promising clinical diagnostic and therapeutic target for some types of tumors.

## Figures and Tables

**Table 1:** Functional characteristics of circHIPK3 in multiple human cancers

Cancer types	Expression pattern	Sponged microRNA	Target genes	Function	Reference
Oral Squamous Cell Carcinoma	Up	miR-124	Unknown	Activate	18
		miR-637	NUPR1	Activate	19
		miR-381-3p	YAP1	Activate	20
Nasopharyngeal carcinoma	Up	miR-4288	ELF3	Activate	22
Gastric cancer	Up	miR-876-5p	PIK3R1	Activate	26
		miR-107	BDNF	Activate	27
		miR-124	Unknown	Activate	28
		miR-29b	Unknown	Activate	28
		miR-653-5p	NRP1	Activate	29
		miR-338-3p	NRP1	Activate	29
		miR-637	AKT1	Activate	30
Lung Cancer	Up	miR-149	FOXM1	Activate	32
		miR-379	IGF1	Activate	33
		miR-124-3p	STAT3	Activate	34
		miR-107	BDNF	Activate	35
Hepatocellular carcinoma	Up	miR-124	AQP3	Activate	38
			PDK2	Activate	39
		miR-506	PDK2	Activate	39
		miR-124-3p	MRP4	Activate	40
		miR-4524-5p	MRP4	Activate	40
		miR-338-3p	ZEB2	Activate	41
Colorectal Cancer	Up	miR-1207-5p	FMNL2	Activate	44
		miR-7	FAK, IGF1R, EGFR, YY1	Activate	45
		miR-637	STAT3	Activate	80
Pancreatic Cancer	Up	miR-330-5p	RASSF1	Activate	47
Prostate Cancer	Up	miR-338-3p	ADAM17	Activate	50
			Cdc25b, Cdc2	Activate	51
		miR-193a-3p	MCL1	Activate	52
		miR-448	MTDH	Activate	53
		miR-212	BMI-1	Activate	54
Melanoma	Up	miR-215-5p	YY1	Activate	57
Glioma	Up	miR-654	IGF2BP3	Activate	59
		miR-124-3p	WEE1 STAT3	Activate	60 62
		miR-124	CCND2	Activate	61
		miR-524-5p	KIF2A	Activate	78
		miR-421	ZIC5	Activate	79
Cervical cancer	Up	miR-338-3p	HIF-1α	Activate	64
		miR-485-3p	FGF2	Activate	65
Cholangiocarcinoma	Up	miR-637	LY6E	Activate	68
Gallbladder cancer	Up	miR-124	ROCK1, CDK6	Activate	70
Esophageal squamous cell carcinoma	Up	miR-599	c-MYC	Activate	71
		miR-124	AKT3	Activate	72
Breast cancer	Up	miR-326	Unknown	Activate	73
		miR-124-3p	MTDH	Activate	75
		miR-193a	HMGB1	Activate	74
		miR-1286	HK2	Activate	81
		miR-582-3p	RNF11	Activate	82
Thyroid cancer	Up	miR-338-3p	RAB23	Activate	76
Chronic myeloid leukemia	Up	miR-124	B4GALT1	Activate	77
Bladder cancer	Down	miR-558	HPSE	Inhibit	83

**Table 2:** Dual expression pattern and functions of circHIPK3 within a same tumor

Cancer types	Expression pattern	Sponged microRNA	Target genes	Function	Reference
Renal Cancer	Up	miR-485-3p	Unknown	Activate	88
	Up	miR-508-3p	CXCL13	Activate	89
	Down	miR-637	Unknown	Activate	90
Osteosarcoma	Up	miR-637	HDAC4	Activate	91
	Up	miR-637	STAT3	Activate	92
	Down	Unknown	Unknown	Inhibit	93
Ovarian cancer	Up	Unknown	Unknown	Unknown	96
	Up	miR-7	VEGF	Activate	97
	Down	Unknown	Unknown	Inhibit	98

**Table 3:** The downstream signal pathways of circHIPK3

Sponged microRNA	Target genes	Signal pathway	Cancer types	Reference
miR-637	NUPR1	PI3K/AKT	oral squamous cell carcinoma	19
miR-381-3p	YAP1	Hippo/YAP	oral squamous cell carcinoma	20
unknown	unknown	Wnt/β-catenin	gastric cancer	25
miR124-3p	STAT3	PRKAA/AMPKα	lung cancer	34
miR-381-3p	unknown	AKT/mTOR	lung cancer	36
miR-7	IGF1R	PI3K/AKT	colorectal cancer	45
miR-7	EGFR	MEK/ERK	colorectal cancer	45
miR-7	YY1	Wnt/β-catenin	colorectal cancer	45
miR-124	AKT3	PI3K/AKT	esophageal squamous cell carcinoma	72
miR-193a	HMGB1	PI3K/AKT	breast cancer	74
miR-338-3p	RAB23	NF-κB; integrin β1/Rac1	thyroid cancer	76
miR-637	STAT3	Bcl-2/Beclin1	colorectal cancer	80

## Abbreviations

circRNAs: circular RNAs
circHIPK3: circRNA homeodomain-interacting protein kinase 3
BC: bladder cancer
CC: cervical cancer
CCA: cholangiocarcinoma
CML: chronic myeloid leukemia
CRC: colorectal cancer
OSCC: oral Squamous Cell Carcinoma
ESCC: esophageal squamous cell carcinoma
EMT: epithelial-mesenchymal transition
RC: renal cancer
OC: ovarian cancer
GBC: gallbladder cancer
GC: gastric cancer
HCC: hepatocellular carcinoma
miRNA: microRNA
MREs: miRNA response elements
NPC: nasopharyngeal carcinoma
NSCLC: non-small cell lung cancer
OS: osteosarcoma
PCa: prostate cancer
PC: pancreatic cancer
CCRCC: clear cell renal cell carcinoma
UTR: untranslated region
ceRNA: competing endogenous RNAs
PTX: paclitaxel
GEM: Gemcitabine
TMZ: Temozolomide
sEV: serum extracellular vesicle
GEM: glioblastoma multiforme
